# Feasibility and acceptability of brief behavioral therapy for cancer-related insomnia: effects on insomnia and circadian rhythm during chemotherapy: a phase II randomised multicentre controlled trial

**DOI:** 10.1038/s41416-018-0154-2

**Published:** 2018-07-20

**Authors:** Oxana Palesh, Caroline Scheiber, Shelli Kesler, Michelle C. Janelsins, Joseph J. Guido, Charles Heckler, Mallory G. Cases, Jessica Miller, Nick G. Chrysson, Karen M. Mustian

**Affiliations:** 1 Department of Psychiatry and Behavioral Sciences, Stanford University, Stanford, CA USA; 2 University of Texas MD Anderson Cancer Center, Houston, TX USA; 3 University of Rochester Medical Center, James P. Wilmot Cancer Institute, Rochester, NY USA; 4 Metro-Minnesota Community Oncology Research Consortium, Minneapolis, MN USA; 5 Novant Health Oncology Specialists, Winston-Salem, NC USA

**Keywords:** Randomized controlled trials, Quality of life

## Abstract

**Background:**

This phase II RCT was conducted to determine the feasibility and acceptability of brief behavioral therapy for cancer-related insomnia (BBT-CI) in breast cancer patients undergoing chemotherapy. We also assessed the preliminary effects of BBT-CI on insomnia and circadian rhythm in comparison to a Healthy Eating Education Learning control condition (HEAL).

**Methods:**

Of the 71 participants recruited, 34 were randomised to receive BBT-CI and 37 to receive HEAL. Oncology staff was trained to deliver the intervention in four community clinics affiliated with the NCI. Insomnia was assessed with the Insomnia Severity Index (ISI), and circadian rhythm was assessed using a wrist-worn actiwatch.

**Results:**

Community staff interveners delivered 72% of the intervention components, with a recruitment rate of 77% and an adherence rate of 73%, meeting acceptability and feasibility benchmarks. Those randomised to BBT-CI improved their ISI scores by 6.3 points compared to a 2.5-point improvement in those randomised to HEAL (*P* = 0.041). Actigraphy data indicated that circadian functioning improved in the BBT-CI arm as compared to the HEAL arm at post-intervention (all *P*-values <0.05).

**Conclusions:**

BBT-CI is an acceptable and feasible intervention that can be delivered directly in the community oncology setting by trained staff. The BBT-CI arm experienced significant improvements in insomnia and circadian rhythm as compared to the control condition.

## Introduction

Sleep problems such as insomnia are prevalent in cancer patients.^1^ Insomnia is defined as difficulty falling or staying asleep or waking up earlier than intended. Insomnia is common in the general population; however, cancer patients are disproportionally affected.^1,2^ In fact, our research suggests that insomnia is two to three times higher in patients undergoing chemotherapy than in the general population.^1^ Precipitating factors for the development of insomnia during chemotherapy include the iatrogenic effects of tumour biology itself, stress associated with diagnosis and treatment, direct effects of chemotherapy, chemotherapy pre-medications (e.g., steroids), and reduction in physical activity.^3^ As a result, cancer patients may be at an increased risk for developing chronic insomnia.^1,3^ Insomnia, associated with many adverse psychiatric and physical side effects, is closely linked with depression, cancer-related fatigue, increased pain, reduced quality of life, disease progression, and survival.^3,4^

Moreover, insomnia can dysregulate circadian rhythm, a 24-h system in part responsible for regulation of the sleep-wake cycle. Robust circadian rhythm is associated with overall good health, while disruption in circadian rhythm is linked to worse overall health outcomes including shorter survival in cancer.^5^ Research suggests that patients undergoing chemotherapy have more severe levels of insomnia than patients receiving other treatments such as surgery, radiation, or hormonal therapy.^6–8^ Thus, given the multiple adverse associations seen with insomnia, it is of particular importance to address insomnia in cancer patients before it becomes chronic.

Two randomised controlled trials (RCTs) have been conducted on behavioral interventions for insomnia in cancer patients during chemotherapy.^9–11^ The first identified clinical trial delivered an energy and sleep enhancement intervention delivered by trained nurses in three telephone sessions, which had no effect on sleep in patients undergoing chemotherapy. In the RCT by Berger et al., patients who participated in a behavioral treatment, Individualized Sleep Promotion Plan, reported significantly better sleep quality, but no improvements in objective sleep were observed.^10,11^ Although Berger and colleague’s intervention was effective in enhancing subjective sleep quality, it required participants’ attendance of six, hour-long face-to-face intervention sessions, and it had a complex, difficult to replicate protocol as participants were allowed to choose strategies of interest. Furthermore, patients were not selected based on the presence of clinical insomnia at baseline, making it difficult to quantify their improvements.

In the general population, cognitive behavioral therapy for insomnia (CBT-I) is considered the gold standard.^12,13^ During sessions, a therapist teaches stimulus control strategies (instructions for behavioral modifications), sleep restriction methods, and cognitive restructuring techniques to address insomnia symptoms. While CBT-I has been shown to be consistently effective for insomnia in cancer patients and survivors,^14–18^ there is a scarcity of research investigating its effects on circadian rhythms (as measured by actigraphy data) in cancer. The limited number of existing studies in patients diagnosed with cancer and cancer survivors utilised actigraphy for sleep measurement but generally did not examine effects of CBT-I on circadian rhythms as measured by actigraphy.^11,15,19,20^

Despite the numerous merits of CBT-I, there are several drawbacks for patients including being very time-consuming (on average, CBT-I consists of seven face-to-face sessions) and costly (requiring a trained professional therapist). Treatment delivery by a professional therapist renders it impractical in many clinical settings. In addition, CBT-I contains elements (i.e., sleep restriction) that might not be practical or safe to implement in patients receiving chemotherapy. To address these limitations and help fill a gap between research and clinical practice, we developed a Brief Behavioral Therapy for Cancer-Related Insomnia (BBT-CI) that is brief, does not require extensive training (can be delivered by clinical staff), and can be conducted in the clinic during while patients are undergoing chemotherapy infusion. Furthermore, BBT-CI, unlike CBT-I, addresses circadian disruption directly by providing education about cancer-associated circadian disruption, adverse effects of circadian disruption on sleep, effects of melatonin on sleep and sleep consolidation, and behavioral instruction on how to entrain circadian rhythms.

We pilot tested BBT-CI at a single academic center to ensure it was acceptable and showed promise for insomnia reduction. We found the intervention to be feasible and acceptable, with medium preliminary effect sizes as compared to a sleep hygiene education brochure. However, feasibility and acceptability of BBT-CI in a multicenter setting with trained clinic staff instead of psychologists were unknown.^21^

The goals of this RCT were to assess the feasibility and acceptability of BBT-CI in a multicenter setting. We wanted first to determine whether cancer patients undergoing chemotherapy would be willing to participate in BBT-CI. Second, we wanted to ascertain whether we could train nurses and clinical research assistants (CRAs) to deliver BBT-CI accurately, and finally, we wanted to establish whether conducting BBT-CI in the community clinics would be feasible. Our secondary aims were to establish the preliminary efficacy of BBT-CI on insomnia and circadian rhythms. Overall, we hypothesised that BBT-CI would be a feasible and acceptable intervention deliverable by trained nurses and CRAs to breast cancer patients actively undergoing chemotherapy. Furthermore, we hypothesised that BBT-CI would show superior preliminary efficacy in the treatment of insomnia symptoms and circadian dysregulation as compared to the control condition.

## Methods

### Sample

Participants were recruited at four University of Rochester Cancer Center (URCC) National Cancer Institute (NCI) Community Oncology Research Program (NCORP) Research Base affiliated institutions. No participant accrual took place at the URCC NCORP Research Base itself. Seventy-one breast cancer patients receiving chemotherapy were randomised to either BBT-CI or a Healthy Eating EducAtion Learning (HEAL) control arm.

Eligible patients met the following criteria: (1) at least 21 years of age; (2) female with newly diagnosed stage I–III breast cancer; (3) undergoing chemotherapy in either weekly, bimonthly, or 3-week cycles with at least 6 weeks of chemotherapy remaining at the time of study enrollment; (4) a score of eight or more on the Insomnia Severity Index (ISI); (5) beginning or worsening of sleep disturbance since cancer diagnosis (did your sleep problems begin or get worse with the diagnosis of cancer or with chemotherapy?); (6) English-speaking; and (7) not using a daily sleep aid except melatonin (use of a sleep aid as needed was permitted, and use was noted).

Patients were excluded who: (1) had a diagnosis of stage IV breast cancer; (2) had sleep problems that began prior to their cancer diagnosis; (3) suffered from another sleep disorder (e.g., sleep apnea, restless leg syndrome); (4) had a history of severe mental illness; (5) were unable to abstain from anxiolytics for 4 h before intervention sessions; (6) had a device implanted due to an existing heart condition; or (7) were shift workers and had irregular sleep-wake cycles due to inconsistent work schedules.

The study was conducted after approval by each center’s institutional review board. Informed written consent was obtained from each participant in this study.

### Randomisation

Participants were randomly assigned using a 1:1 ratio of the BBT-CI intervention or HEAL control condition. Randomisation was stratified by NCORP site and ISI score obtained during the screening process. Participants were divided into those with moderate sleep problems (ISI score of 8–15 = “moderate”) and those with severe sleep problems (ISI scores of 16–28 = “severe”). These cutoffs, based on ISI’s manual recommendations,^22^ were determined prior to the study. Approximately an equal number of participants with moderate to severe sleep problems were randomised into each intervention arm.

#### BBT-CI intervention arm

The proposed BBT-CI intervention was, in part, modeled on standard CBT-I. BBT-CI includes both stimulus control and sleep scheduling; however, it was modified from CBT-I to make it more suitable for cancer patients actively undergoing chemotherapy with recently developed sleep problems. The primary intervention component consisted of one 60-min face-to-face session with a trained NCORP staff member during which time an individually tailored treatment plan was developed. There were also four 15-min phone calls and a second 60-min face-to-face “booster” session. The second face-to-face session occurred 3 or 4 weeks following the initial session, depending on the participant’s chemotherapy regimen. The face-to-face sessions were delivered in the clinic during the participant’s chemotherapy appointments to reduce participant burden. If a treatment was missed, or an appointment changed, the second face-to-face session was conducted the following week and documented within the deviations section of the Intervener Therapy Checklist. Assessments were conducted by an NCORP staff member who was not involved in the intervention to minimise bias.

The BBT-CI intervention included the following five components: (1) education: at the beginning of the intervention, the Spielman model of insomnia^23,24^ was reviewed with the participant, followed by a discussion on how insomnia may co-occur and interact with cancer and its treatments. Education about the contribution of circadian disruption to insomnia was also provided. (2) Stimulus control: the participant was encouraged to reserve in-bed activities to sleep and sex only. Furthermore, the participant was prompted not to go to bed until sleepy, to wake up at the same time every day, and to get out of bed if unable to fall asleep within 15–20 min. (3) Discouragement/modification of napping: this part of the intervention included education on napping. The participant was encouraged not to nap or, if napping could not be avoided, to limit napping to two naps per day with a duration of no longer than 45 min. The participant was told they could nap and sleep as much as needed on post-chemotherapy days. (4) Sleep compression: the participant was encouraged to postpone her bedtime by at least 15 min if sleep efficiency was <90%. Sleep efficiency was defined as total sleep time/time in bed × 100%. Given the intervention’s brevity, we wanted to capture participants beginning to develop insomnia issues in addition to those with existing insomnia and, for that reason, chose a more stringent sleep efficiency cutoff. (5) Chronorehabilitation: sleep-wake cycles were regulated by encouraging the entrainment of circadian rhythm and sleep-wake cycles. Furthermore, the participant was encouraged to increase bright light exposure during the day, decrease it during the night, and increase physical activity levels in general.

#### HEAL control arm

The HEAL control arm was matched to the BBT-CI intervention arm on time and attention but had no behavioral elements related to sleep. The control arm was patterned based on the RCT conducted by Berger et al.^10,11,25^ The control intervention provided an alternative treatment with precisely the same schedule as the BBT-CI treatment arm, with two face-to-face sessions and four phone calls. The content of the control intervention was provided by the NCI. The topics included nutritional implications of chemotherapy, nutrition screening and assessment, oral nourishment, and nutritional suggestions for symptom management (anorexia, alterations of taste and smell, xerostomia, mucositis/stomatitis, nausea, diarrhoea, neutropaenia, hydration and dehydration, and constipation).

### Interveners training

At least two staff members from each of the four participating sites were selected to deliver the intervention (i.e., the interveners). Eligible interveners had at least a college degree and 1 year of direct patient experience in oncology clinics. Over the four treatment sites, we had 16 interveners who received an average of 12 training sessions.

Eligible interveners were trained directly by the principal investigator, Dr. Oxana Palesh, and her designated research staff over the phone. Additional training included: reading the manual and other web-based resources that were posted on the secure NCORP website, watching the training videos, and role-playing with peers. To warrant fidelity of the study results, we ensured that each intervener received the appropriate training before working with participants. Once the training was completed, an intervener conducted a scheduled practice session with Dr. Palesh or her staff, who provided feedback and either certified them to deliver the intervention or scheduled additional practice to improve their competency and completed another certification session. Both written and oral feedback was provided to each intervener to hone skills. Every initial face-to-face session was recorded and reviewed for adherence to the intervention protocol. In addition, a checklist of adherence to BBT-CI protocol consisting of the essential intervention elements was completed by both the intervener and participant.

### Measures

Participants completed several physiological and symptom outcome measures at baseline and post-intervention. What follows is a description of subjective insomnia and objective circadian rhythm measures used in subsequent analyses.

### Primary outcome

Feasibility and acceptability were measured with screening logs and intervention checklists. Recruitment of the participants was documented at each NCORP site via screening logs. Participants’ adherence was assessed with attendance logs that were completed by the interveners at each of the sites. BBT-CI participant feedback of the intervention was assessed at the end of the post-intervention follow-up visit via two questions: (1) “How useful do you think BBT-CI was in helping sleep quality?” answered on a Likert scale of 1—“Very” to 4—“Doesn’t seem to help” and (2) “Based upon your experience with BBT-CI, would you recommend it to other cancer patients?” answered on a Likert scale of 0—“Strongly Do Not Recommend” to 4—“Highly Recommend.” To assess the feasibility and acceptability of training nurses and CRAs to deliver the BBT-CI intervention’s components correctly, we developed an intervener therapy checklist and participant therapy checklist that were completed by the intervener and participant separately after each session. Both questionnaires used a three-point scale to rate the degree to which specific core intervention components were delivered during each session. Each intervention component was scored as (1) covered fully, (2) covered partially, or (3) not covered at all.

The intervener checklist also included a section documenting the environment where the intervention was conducted, describing the following settings: private room versus open space, multi-purpose room, with or without distractions. Additionally, to assess the treatment protocol adherence, the two face-to-face sessions were audio recorded, securely stored, and reviewed by the principal investigator and her designated research staff within 72 h of session completion. Interveners received feedback prior to each subsequent session based on the evaluation of the recorded session to strengthen adherence. At the conclusion of the study, each participant also completed a feedback questionnaire to assess the acceptability of the intervention.

### Secondary outcomes

#### Insomnia

The secondary outcome measure for the study was the ISI, a valid and reliable self-report questionnaire of sleep.^26^ The ISI consists of seven sleep-related questions ranked on a five-point Likert scale. Individual item scores are summed to acquire a total score ranging from 0 to 28. The measure takes ~5 min to complete.

#### Circadian rhythm

In addition to the insomnia outcome, we also used an objective marker of circadian rhythm functioning. Circadian rhythm functioning was estimated with the two-oscillator cosinor model (12- and 24-h period) based on the actigraphy data. Actigraphy data were measured with the wrist-worn Actiwatch-64 (MiniMitter, Bend, OR). The Actiwatch-64 monitored movement as well as light exposure. The actiwatch was configured to record activity counts every 60 s. Participants were asked to wear the actiwatch 1 week before the intervention and 1 week before each follow-up visit.

Actigraphy data were analysed with the Actiware^®^ software (v.5.04.003, MiniMitter, Bend OR) by the PEAK Laboratory at the URCC NCORP Research Base. The software program uses sleep algorithms to calculate “in-bed/out-of-bed” portions of the day, thereby, assessing the percentage of sleep acquired, nighttime sleep patterns, as well as sleep quality.

The actiwatch measured the following domains of circadian rhythm: (1) mesor, a mean score adjusted by the dips and rises of the circadian rhythm that is indicative of overall circadian rhythm function; (2) amplitude at 12- and 24-h cycles, a difference score between the mean value of the circadian rhythm wave and its peak; and, (3) acrophase at 12- and 24-h cycles, the time of day associated with peak activity.

### Statistical analysis

#### Power analysis

The power analysis based on the feasibility and acceptability outcomes revealed that we needed 30 subjects per arm to reach power of 80% or greater, with a one-sided test at the 5% significance level. We expected an attrition rate of 15%. As a result, we aimed to recruit 35 participants per treatment arm.

#### Analytical strategy

The following three aims served as benchmarks of feasibility and acceptability: (1) 40% or more of eligible patients consent to participate in the study, (2) at least 75% of the consented participants complete at least five intervention sessions, and (3) at least 80% of the key intervention components, as assessed by checklists and audio recordings, are delivered by the interveners. On average, 85% of CBT participants in a traditional setting (longer-term therapy, not necessarily medically ill) attend all of their intervention sessions and complete the majority (>80%) of the treatment components.^27^ In our study, slightly lower benchmark of acceptability at 75% of participants completing at least five out of six sessions (83.3%) was set because our population was undergoing medical care, was in a community setting, and has a high burden of comorbidities making pharmacological interventions for insomnia less desirable.

Aims 1 and 2 were evaluated with a binomial test with the probability of success denoted as *P* = 0.40 and *P* = 0.75, respectively. *χ*^2^ tests were used to analyse whether rates of consent and completion of study procedures differed between the two treatment arms. Aim 3 was assessed by calculating and testing the overall mean percent of delivery using random effect modeling (REML estimation) with the Kenward–Roger procedure. We set the significance value at 0.017 due to the three assessments that needed to be conducted (Bonferroni correction, 0.05/3 = 0.017).

Analyses of covariance (ANCOVAs), *t*-tests, and nonlinear regression models were used to assess group differences in insomnia and circadian rhythm outcome measures between the treatment arms at baseline and post-intervention. Change scores (post-intervention minus pre-intervention scores) were examined depending on the correlation between the two time points’ scores of interest. When the correlation was less than *r* = 0.50, a *t*-test was used, given that *t*-tests have more power than ANCOVAs.^28^ When the correlation was *r* = 0.50 or greater, an ANCOVA was used. For ANCOVAs, the main effect included the two intervention arms (BBT-CI, HEAL), the baseline scores served as a covariate, and the post-intervention scores served as a dependent variable.

Differences in circadian rhythm functioning between the two arms were analysed with ANCOVA on the two-oscillator cosine model (12- and 24-h cycles). Circadian parameter estimates obtained were calculated from the log (1 + activity counts) of the actigraphy data (mesor, amplitude for the 12- and 24-h cycles, and acrophase for the 12- and 24-h cycles).

#### Missing data

Once we established the reasons for dropout from our records, we could infer that the data were missing at random. We used multiple imputations to establish that the imputed analyses were not substantively different from the analysis of complete cases (reported here).^29,30^

## Results

### Participants

In total, 71 patients participated in the study (mean age = 53 years, SD = 9.8). Most participants were enrolled in cycle 2 or 3 of chemotherapy, meaning they were ~2 months from diagnosis. Thirty-four women were randomised to receive the BBT-CI treatment intervention (mean age = 51 years, SD = 7.9) and 37 women were randomised to receive the HEAL control condition (mean age = 54 years, SD = 11.2). Participants assigned to the HEAL control arm did not receive the BBT-CI intervention at the conclusion of the study.

There were no significant differences in demographic or medical status variables at baseline between the two arms, including sleep disturbance severity and baseline ISI scores. An equal number of women with stage I cancer was present in both intervention arms, (21% and 18%, respectively). In the BBT-CI arm, 20 women (58%) had stage II breast cancer (58%), and 7 had stage III (21%), as compared to 19 (52%) and 9 (25%) respectively in the HEAL arm. Group differences were not significant (*P* > 0.05). See Table 1 for more details on demographic and medical characteristics of the sample.

**Table 1:** Demographics table for full sample (*N* = 71): Brief Behavioral Intervention for Cancer-Related Insomnia arm (*n* = 34) and Healthy Eating Education Learning control condition (*n* = 37)

	Total	BBT-CI	HEAL
*Demographic variables*			
Gender			
Female, *N* (%)	71 (100%)	34 (48%)	37 (52%)
Age, mean (SD)	52.5 (9.78)	50.9 (7.91)	53.8 (11.17)
Race, *N* (%)			
White	68 (96%)	33 (97%)	35 (96%)
Black	2 (3%)	1 (3%)	1 (2%)
Asian	1 (1%)	0 (0%)	1 (2%)
Ethnicity, *N* (%)			
Hispanic or Latino	0 (0%)	0 (0%)	0 (0%)
Non-Hispanic	71(100%)	34 (100%)	37 (100%)
Marital status, *N* (%)			
Married/long-term partner	48 (68%)	28 (83%)	20 (56%)
Divorced	11 (15%)	3 (9%)	8 (22%)
Single	4 (6%)	2 (7%)	2 (5%)
Widowed	1 (1%)	0 (0%)	1 (1%)
No answer	7 (10%)	1 (1%)	6 (16%)
*Medical and symptom variables*			
Stages of disease, *N* (%)			
Stage I	14 (20%)	7 (21%)	7 (18%)
Stage II	39 (54%)	20 (58%)	19 (52%)
Stage III	16 (23%)	7 (21%)	9 (25%)
Unknown	2 (3%)	0 (0%)	2 (5%)
Time since diagnosis, mean days (SD)	74.01 (31.23)	72.00 (26.91)	75.97 (35.20)
Sleep disturbance severity, mean (SD)			
Prior to cancer diagnosis	3.5 (2.16)	3.6 (2.12)	3.4 (2.23)
Since cancer diagnosis	6.9 (1.51)	7.0 (1.59)	6.7 (1.42)
Last 7 days	6.7 (1.72)	6.8 (1.65)	6.6 (1.82)
Entry ISI, *N* (%)			
Moderate	36 (51%)	16 (47%)	20 (55%)
Severe	35 (49%)	18 (53%)	17 (45%)
Usage of sleep aids, *N* (%)			
Yes	30 (43%)	18 (53%)	12 (32%)
No	29 (41%)	14 (41%)	15 (41%)
No answer	12 (16%)	2 (6%)	10 (27%)

Sleep disturbance severity was measured via a three-item self-report measure “Please rate the severity of your problems with sleep prior to your cancer diagnosis/since your cancer diagnosis/in the last 7 days” on a Likert scale (1 = “Not at all” to 10 = “As bad as you can imagine”). This three-item measure was given to participants at the baseline study visit and thus asked retrospectively

### Recruitment, acceptability, and feasibility

**Fig. 1:**
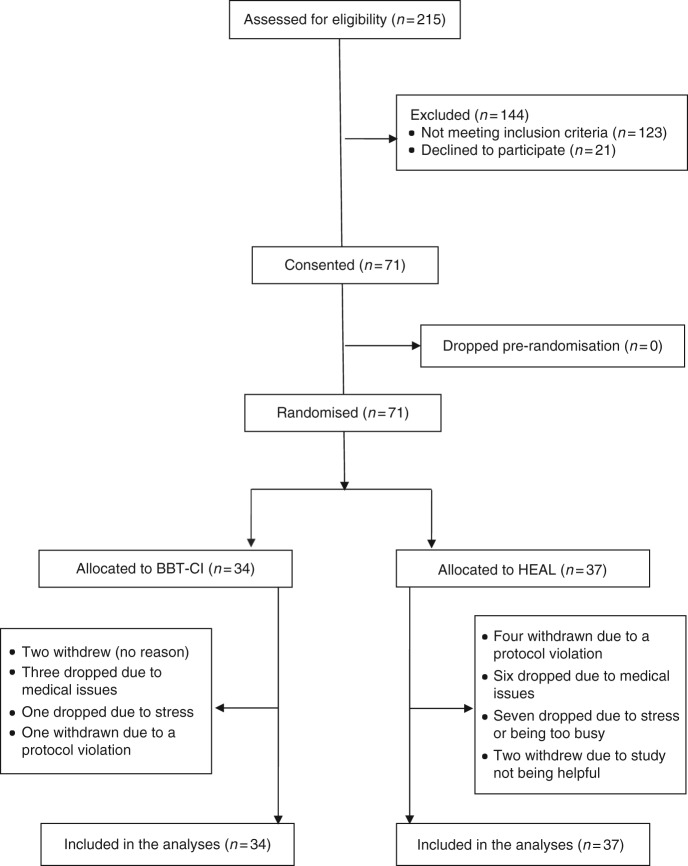
CONSORT diagram depicting participant numbers from recruitment phase through completed cases included in final analyses

Figure 1 is a CONSORT diagram summarising the flow of participants through the study. We had four recruitment sites: Metro Minnesota, MN; Wichita, KS; Hematology Oncology Associates, NY; and Southeast Cancer Control Consortium (SCCC, comprising VA, NC, SC, and GA). Recruitment began in February 2014 and ended in December 2015. In total, 92 patients were eligible to participate, 21 of which declined to participate due to lack of interest in the study. Seventy-one eligible cancer patients agreed to participate in the study, reflecting a recruitment rate of 77.2% (CI = 67.6%–84.6%). In the intervention arm (BBT-CI), 25 of the 34 participants completed at least five of the six BBT-CI sessions, reflecting an adherence rate of 73.5% (CI = 55.3%–86.5%). Furthermore, review of checklists and audio recordings verified that interveners successfully delivered 80.7% of the intervention components (CI = 58.8%–100.0%). The intervention feedback questions indicated that BBT-CI participants, post-intervention, thought the BBT-CI intervention helped sleep quality (mean = 1.46, SD = 0.58) and, based on their experience, would recommend it to other cancer patients (mean = 3.21, SD = 0.92).

### Insomnia and circadian rhythm outcomes

**Fig. 2:**
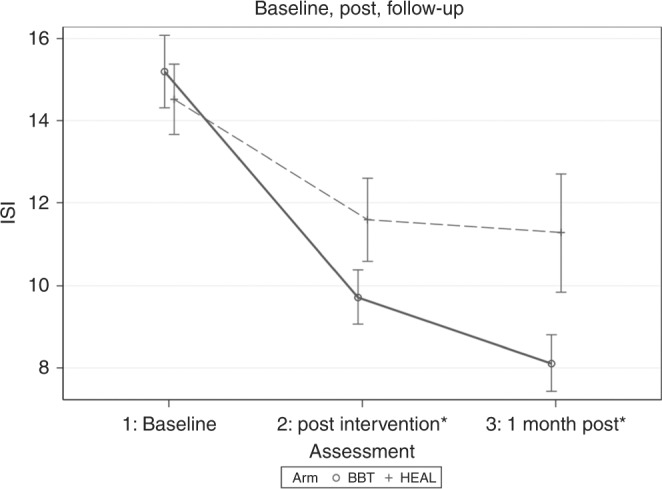
Changes in mean sleep disruption as measured by ISI at baseline, post-intervention, and 1-month follow-up for the two treatment arms: BBT-CI (solid black line) and HEAL (dotted gray line). Error bars represent 95% confidence intervals. ISI Insomnia Severity Index, BBT-CI Brief Behavioral Treatment for Cancer-Related Insomnia, HEAL Healthy Eating Education Learning Control Condition (*between-group differences, *P* < 0.05)

For the ISI, a significant effect was found in favour of the BBT-CI treatment arm at post-intervention (*t* = −2.12, *P* = 0.041). The BBT-CI intervention arm’s ISI total score decreased by a mean of 6.3 points between baseline and post-intervention (SD = 5.1) as compared to 2.5 points for the HEAL control condition (SD = 6.7). Results are shown in Fig. 2.

For the circadian rhythm parameters (mesor, 12- and 24-h amplitude, and 12- and 24-h acrophase), we found no significant group differences at baseline (*P* > 0.05); however, we did find a significant group-by-baseline interaction for mesor (*t* = −2.710, *P* = 0.010) and a main effect (*t* = 2.861, *P* = 0.007). Participants in the BBT-CI arm who had low baseline mesor (e.g., average activity level) showed higher mesor (mean activity) at post-intervention. At post-intervention, we also found significant effects in favour of those randomised to the BBT-CI arm for 24-h amplitude (*t* = 3.971, *P* = 0.0003), with BBT-CI participants having a higher amplitude than the participants in the HEAL control condition. A significant group-by-baseline interaction was found for 12-h acrophase (*t* = 2.287, *P* = 0.027) with a significant main effect (*t* = −2.420, *P* = 0.020). BBT-CI participants had a smaller 12-h acrophase than HEAL participants when baseline acrophase was less than the median (17.5). No effects were found for 12-h amplitude nor group effects for 24-h acrophase at post-intervention.

### Adverse events

No serious adverse events (AEs) occurred during the study. No participants randomised to the BBT-CI arm reported any AEs. Three HEAL participants experienced seven mild AEs (e.g., nervousness, agitation, headache). There were no AEs that could be specifically attributed to the study or the study’s intervention.

## Discussion

BBT-CI was found to be an acceptable intervention, as shown by high recruitment and adherence rates (77.2% and 73.5%, respectively). Furthermore, trained nurses and CRAs successfully delivered at least 75.0% of the intervention components, providing evidence for its feasibility. BBT-CI can be learned by staff who are not professional therapists, suggesting that the intervention is practical to deliver in community cancer clinics, which tend to have limited access to professional psychological services.

In addition, BBT-CI showed efficacy in reducing insomnia symptoms compared to the HEAL nutrition control condition in breast cancer patients undergoing chemotherapy. These findings are clinically relevant, given that the vast majority of patients undergoing chemotherapy experience acute symptoms of sleep disruption.^1,8^ While some patients will recover from their sleep difficulties, many will develop chronic problems of insomnia later.^1,8^ This underscores the importance of treating sleep disruption in cancer patients while it is acute to prevent the development of chronic insomnia.

In addition to the insomnia findings, we also found significant group differences in circadian rhythm physiology. The actigraphy results showed that participants randomised to BBT-CI experienced higher overall mean activity (mesor), higher peak to nadir of activity difference (amplitude), and smaller acrophase (earlier peak of activity) compared to participants randomised to HEAL. The significant changes in circadian rhythm for those randomised to BBT-CI may be viewed as improvement since they were in concordance with subjective improvements in insomnia and were in the direction supported by prior research.^31^ Although disruption of the sleep-wake cycle and circadian rhythm do not always co-occur, chronic sleep-wake cycle disruption can eventually dysregulate circadian rhythm, and conversely, long-term circadian rhythm disruption can result in chronic sleep disruption (i.e., insomnia).^32^

Reduction in insomnia can lead to improvements in the circadian rhythm by synchronising circadian rhythm with the sleep-wake cycle. Improving circadian rhythm is of high importance as circadian rhythm disruption has been implicated in cancer progression.^5,33,34^ In fact, the World Health Organization considers shift work, work taking place outside the traditional 9 am–5 pm shift, to be carcinogenic because it disrupts circadian rhythms.^5,35,36^ To our knowledge, this is the first RCT to show improvements in circadian rhythm as a result of a brief behavioral sleep intervention among breast cancer patients undergoing chemotherapy.

Previous studies using CBT-I did not find an impact on circadian rhythmicity because CBT-I is not designed to address it. However, one of the key differences between CBT-I and BBT-CI is that BBT-CI focuses on circadian rhythm disruption through education and behavioral instruction. While we cannot be entirely certain, without dismantling our intervention, that these differential components influence circadian rhythm, we believe the addition of these elements accounts for the circadian effects seen in this trial.

Our findings are significant in light of data consistently showing that face-to-face CBT-I interventions outperform CBT-I delivered via booklets, self-guided, or in video format. Having a face-to-face intervention that is shorter than traditional CBT-I, yet still effective, is important. An RCT employing a video-based CBT-I treatment in 242 patients with breast cancer provided evidence for the efficacy of a video-based CBT-I intervention in patients with breast cancer but revealed that face-to-face therapy interventions show superior treatment effects.^18,37^ Another RCT compared an early minimal CBT-I booklet intervention to a no-treatment control condition in 38 cancer patients. While the booklet showed efficacy, the study was lacking a comparison with face-to-face therapy.^38^ Additionally, two RCTs, both showing improvements in insomnia symptoms, utilised an internet intervention^ 39^ and a web-based intervention ^40^ in cancer survivors, not patients actively undergoing treatment, with insomnia; however, neither compared the intervention to face-to-face therapy. Given these results, it is likely that face-to-face interventions, like BBT-CI, are more efficacious in treating insomnia in breast cancer patients. In sum, our present study compared BBT-CI, a face-to-face treatment intervention, with HEAL, a treatment control condition, and demonstrated excellent preliminary efficacy of BBT-CI in treating insomnia in breast cancer patients actively undergoing chemotherapy.

One of the many strengths of our design is that it allows us to capture patients as they are developing insomnia symptoms, but before problems become chronic and require more intensive treatment. BBT-CI is innovative because it is the first behavioral intervention to treat insomnia at the bedside in the infusion center, and it can be used in tandem with biomedical treatments. This unique feature is a focal point of translational behavioral research as it minimises patient burden and thereby provides guidelines for a new paradigm of care delivery. Findings suggest that behavioral interventions can be made readily available to patients in the infusion clinic and can be delivered by trained hospital staff. In addition, this treatment modality may be feasible for the treatment of other prevalent side effects from which cancer patients suffer, such as depression, anxiety, fatigue, or cognitive difficulties.^6^ There is a need to develop non-pharmacological interventions that are portable and accessible to many patients during treatment.

Another important aspect of this research revolves around the integration of behavioral health services in medical settings. Research has provided evidence that the delivery of behavioral health services in medical clinics may reduce morbidity and mortality and can increase cost-effectiveness overall.^41^ Indeed, the interconnectedness between behavioral health and medicine give rise to the possibility of positive additive effects. For example, treating cancer-related side effects, such as insomnia, fatigue, or anxiety, might also have a positive effect on patients’ adherence to medical treatments.

The study has many strengths including the use of a randomised design, a manualised intervention, and a standardised training protocol which allow for replication of findings. Furthermore, the inclusion of a strong control condition matched for time and attention, the use of a validated self-report measure of insomnia, and objective measurement of sleep and circadian rhythm, as well as the utilisation of different community oncology network clinics across the United States, enhance the generalisability of the results. Despite its strengths, findings of this study need to be interpreted in light of its limitations. The study was designed to recruit only women with non-metastatic breast cancer which may limit its generalisability to other cancers and men. The participants in the study were predominately white and well-educated. As the primary goal was to establish feasibility, the study was relatively small.

A large-scale phase III RCT is needed to replicate the present findings to confirm the effectiveness of BBT-CI. If these findings are replicated, BBT-CI might become part of the standard of care for the treatment of insomnia in breast cancer patients undergoing chemotherapy. Longer-term follow-up visits are also needed to determine the sustainability of insomnia and circadian rhythm improvements associated with BBT-CI. Future studies should consider statistically controlling for confounding variables, such as the type of chemotherapy received, thereby enhancing accurate interpretation of BBT-CI’s causal effects on insomnia symptoms and circadian rhythm functioning. Finally, future research can explore the mechanisms of BBT-CI and its impact on physiological markers, such as inflammation.

In combination, findings provide evidence for the preliminary efficacy of an innovative care delivery model by which trained nurses and research staff in community oncology clinics can deliver a behavioral intervention, successfully reduce insomnia, and improve circadian rhythm functioning among breast cancer patients actively undergoing chemotherapy.

## Availability of data and material

The data supporting the results reported in the article can be requested from the University of Rochester Cancer Center Research Base.
